# Cognitive Science Meets Psychoanalysis

**DOI:** 10.3389/fpsyg.2012.00353

**Published:** 2012-09-26

**Authors:** Todd McGowan

**Affiliations:** ^1^Department of English, University of VermontBurlington, VT, USA

One of the strongest divides that exists in the contemporary intellectual world is that between cognitive science and cultural theory. Cognitive scientists are committed to the idea that we can follow the conceptions of the mind to their zero level of the brain. As a result, speculation about our conceptions on their own terms will always mislead us into false problems. These are the problems that preoccupy cultural theorists. For the latter, cognitive science performs a grand reduction that fails to account for the gap that forever separates the operations of the mind from those of the brain. Cognitive science, in other words, cannot explain the very emergence of mind and thus leaves us on a dead end street.

Few theorists launch themselves into this quarrel without clearly taking position on one side or the other. Even the most sophisticated analysts end up finally refusing to grant the ultimate legitimacy of the opposing side. The cognitive turn within the humanities represents an attempt to bridge the gap, but it does so clearly from the side of cognitive science, even though its practitioners are almost always cultural theorists. What Mark Pizzato has done in *Inner Theatres of Good and Evil: The Mind's Staging of Gods, Angels and Devils* (Pizzato, 2011) and in his previous work *Ghosts of Theatre and Cinema in the Brain* (Pizzato, 2006) is to give equal credit to the discoveries of cognitive science and those of cultural theory, specifically psychoanalysis. This constitutes the singular merit of his contribution, and it would be worth considerable attention if there were it only merit. But there is much more.

Pizzato's concern is not a synthesis of cognitive science and cultural theory in the pseudo-Hegelian sense but rather as a struggle between competing forces. There is thus for Pizzato not just an antagonism within the order of being (as there is for someone like Slavoj Žižek) or an antagonism within the order of society (as there is for someone like Ernesto Laclau) but an antagonism between these two orders. Pizzato's explanation near the opening of the book is worthy of citation. He says, “Species and specific mutations survive because they are better adaptations to their co-evolving environment, including the other species around them. However, humans have evolved the ability to radically transform their environment, as well as their own brains, through certain cultures and technologies – vastly increasing the rate of change in material and virtual realities, beyond nature's balancing acts and incremental adjustments” (Pizzato, 2011, p3). This claim establishes not a symbiotic or homeostatic relation between cognition and culture but an antagonistic one. The project of culture, as Pizzato establishes it, is to fight against the nefarious aspects that the evolution of our brain bequeaths to us. As his history of the brain's performances indicates, sometimes culture is up to the task, and sometimes it is not.

In this present work, Pizzato displays how the notions of good and evil develop through the brain's evolution and manifest themselves through the performances of deities (both good and evil). He traces the theater of the brain from its first recording in prehistoric cave art all the way through recent popular films (like *City of Angels* and *The Passion of the Christ*). Throughout all of these discussions, Pizzato's concern is with the position that God and the Devil take on the brain's stage. The need to create performances involving such figures, Pizzato argues, stems initially from the evolutionary struggle for survival. But the performance itself, the theater, then plays with this evolutionary mandate in a variety of ways. This is where the true focus of the book lies.

Pizzato traces the various depictions of gods and devils in order to understand how the human subject is struggling with its evolutionary heritage. The subject matter that the book addresses is immense. Though he begins briefly with early cave painting, Pizzato spends considerable time on many different forms of theater – from the Medieval *Everyman* to the Renaissance *Hamlet* to the Realist *Hedda Gabbler*. He devotes the concluding chapter to cinematic gods and devils, with an especially compelling discussion of Mel Gibson's *Passion of the Christ*. Rather than simply dismissing the film as a fundamentalist tract, Pizzato nicely analyzes its complexity and the reminder that it offers to us about the permanence of sacrifice.

Pizzato is a thinker well grounded in the thought of Jacques Lacan. He is responsible for many important works in the renaissance of Lacan's thought that occurred in the 1990s and early 2000s. What stands out about *Inner Theatres of Good and Evil* is Pizzato's ability to call on his background in Lacan's thought and to facilitate an encounter between Lacan and cognitive science. Though there is a strong divide among Lacanian thinkers on this subject, Pizzato is a compatibilist. That is to say, he believes that Lacan registers of the symbolic, imaginary, and real correspond to the specific aspects of the brain's anatomy. This belief enables him to make an important contribution to both the expansion of the cognitive sciences into the humanities and to the advancement of Lacanian theory. Anyone seeking to find a way to reconcile these two seemingly disparate intellectual paths would not go wrong immersing herself or himself in Pizzato's *Inner Theatres of Good and Evil*.
